# Development of a semi-empirical physical model for transient NO_x_ emissions prediction from a high-speed diesel engine

**DOI:** 10.1177/14680874241255165

**Published:** 2024-06-18

**Authors:** Abdullah Bajwa, Gongyi Zou, Fengyu Zhong, Xiaohang Fang, Felix Leach, Martin Davy

**Affiliations:** 1 Department of Engineering Science, University of Oxford, Oxford, UK; 2 Department of Mechanical & Manufacturing Engineering, Schulich School of Engineering, University of Calgary, Calgary, AB, Canada

**Keywords:** Semi-empirical NOx model, artificial neural networks, internal combustion engines, diesel, NO_x_, transient, crank-angle resolved NO_x_

## Abstract

With emissions regulations becoming increasingly restrictive and the advent of real driving emissions limits, control of engine-out NO_x_ emissions remains an important research topic for diesel engines. Progress in experimental engine development and computational modelling has led to the generation of a large amount of high-fidelity emissions and in-cylinder data, making it attractive to use data-driven emissions prediction and control models. While pure data-driven methods have shown robustness in NO_x_ prediction during steady-state engine operation, deficiencies are found under transient operation and at engine conditions far outside the training range. Therefore, physics-based, mean value models that capture cyclic-level changes in in-cylinder thermo-chemical properties appear as an attractive option for transient NO_x_ emissions modelling. Previous experimental studies have highlighted the existence of a very strong correlation between peak cylinder pressure and cyclic NO_x_ emissions. In this study, a cyclic peak pressure-based semi-empirical NO_x_ prediction model is developed. The model is calibrated using high-speed NO and NO_2_ emissions measurements during transient engine operation and then tested under different transient operating conditions. The transient performance of the physical model is compared to that of a previously developed data-driven (artificial neural network) model, and is found to be superior, with a better dynamic response and low (<10%) errors. The results shown in this study are encouraging for the use of such models as virtual sensors for real-time emissions monitoring and as complimentary models for future physics-guided neural network development.

## Introduction

The road transport sector accounts for around 16% of global greenhouse gas (mostly CO_2_) emissions.^1^ There has been a strong push towards powertrain electrification in the last decade in order to reduce its CO_2_ footprint. However, because of its high efficiency and superior torque performance, the diesel internal combustion engine is expected to continue being an important propulsion unit, especially for heavy-duty vehicles.^2^ Therefore, it is necessary that efforts to make its combustion clean, that is, reduce exhaust pollutant emissions, continue. A major pollutant species from diesel engines are the oxides of nitrogen (NO_x_), which require substantial reduction to comply with upcoming emissions regulations.^3^ Moreover, with the advent of ‘real driving emissions’ (RDE) based regulations, dynamic control of NO_x_ emissions during transient operation has become important.

NO_x_ emissions from diesel engines can be managed either by controlling in-cylinder processes to lower combustion temperature (e.g. via mixture enleanment or dilution) or via exhaust after-treatment (e.g. selective catalytic reduction).^1^ For dynamic NO_x_ control, real-time engine-out NO_x_ emissions data is needed to permit timely operational adjustments. Closed-loop NO_x_ control using exhaust sensors is impractical because such sensors are expensive and are limited by their dynamic response, which is in the order of seconds.^4^ Cyclic-level engine control requires information in the order of milliseconds. Alternatively, NO_x_ emissions can be predicted from engine models (so-called ‘virtual sensors’^5,6^) and used for ‘model-based control’.

NO_x_ prediction models can be divided into three broad categories: phenomenological (also called physics-based or physical) models,^7–9^ semi-empirical (or mean value) models,^10–12^ and empirical models. Phenomenological models use governing equations that capture the thermochemical and fluid mechanical phenomena taking place during engine combustion and are spatially resolved (e.g. multi-zone thermodynamic or 3D CFD models^13,14^). They thus have high accuracy and predictive strength (accuracy at off-design conditions) but can be computationally expensive. Empirical models use emissions look-up tables or correlations developed from engine calibration testing. They have fast response rates but perform poorly under off-design conditions. A new category of empirical models that have become popular in recent decades are so-called data-driven models. Semi-empirical models use mathematical formulations based on idealised engine combustion and NO_x_ production sub-models to predict general NO_x_ production behaviour. The resulting estimates faithfully capture trends across different engine operating conditions but are limited in their accuracy because of deviations from the ideal NO_x_ production mechanisms assumed. This is mitigated by calibrating the model against experimental data using tuning (or correction) factors. Semi-empirical models offer a good balance between accuracy, predictive power and computational expense, which makes them suitable for dynamic engine control.

This paper compares the transient performance of a semi-empirical NO_x_ prediction model to a previously developed purely data-driven model^15^ for a high-speed diesel engine. A brief literature survey of both the modelling frameworks is presented next.

### Data-driven NO_x_ models

Data-driven models take advantage of the increased availability of engine performance and emissions data. They use large volumes of empirical data to ‘train’ prediction models using different machine learning techniques. Data-driven models are different from traditional empirical models in that they do not require a priori information about the process relationship, which is often prescribed through the form of mathematical correlations that are calibrated to fit experimental data. Rather, only general instructions about the input and output arguments are provided and the model uses machine learning techniques to come up with suitable structures. This makes them an enticing choice for NO_x_ prediction, which depends on multiple, non-linearly related engine combustion and operating parameters, including but not limited to intake air properties, injection settings, trapped mixture composition (air-fuel ratio, internal and external EGR) and mixedness levels. In recent years, such emission models have gained significant interest within the engine development community. A variety of machine learning techniques have been used within the community including gradient boosting techniques,^16^ random forests^17^ and physics aware machine learning techniques.^18^ The machine learning implementation approach of Artificial Neural Networks (ANN) also referred to as deep learning or simply neural networks has been a predictive tool of choice for engine researchers because of its suitability for modelling complex, non-linear processes of the sort that take place during combustion.^19^ ANN models capture non-linear effects through the activation function linking neurons in the hidden and output layers.

Various studies have used the artificial neural networks approach for the prediction of engine performances and emissions. Parlak et al.^20^ demonstrated their ANN model in predicting specific fuel consumption and exhaust temperature with fast and consistent results featuring a low absolute relative error compared to the experiment. Fang et al.^21^ highlighted the importance of ANN input feature selection where the ANN with Pearson correlation selected features demonstrated improved prediction in the low-NO_x_ region. Applying the same model structure, Fang et al.^15^ further extended the ANN model to predict cyclic transient NO_x_ emissions. While the model was able to capture the critical correlation of in-cylinder peak pressure in transient NO_x_ formation in both transient load step-up and step-down, a deviation was observed for all tested load step-down conditions suggesting additional experimental data and/or details of the underlying physical phenomena need to be included under these conditions. This is the baseline ANN model used in the current work. Similarly, the model constructed by Di Mauro et al.^19^ was found to be able to predict the indicated mean effective pressure (IMEP) and its coefficient of variation (CoV) in a spark-ignited internal combustion engine discovering a strong correlation between the modelled CoV and the experiments. However, a systematic overprediction of CoV was observed for low CoVs while higher CoVs were underpredicted by the ANN model suggesting missing physical parameters for the ANN input features. More recently in order to overcome some of the above-mentioned challenges, super learner-based ensemble machine learning approaches were developed for rapid engine design optimisation targeting better fuel efficiency and lower emissions^22–24^

From the above-mentioned studies, the success of data-driven models is found to rely on high-fidelity training data where the optimised models are found fast and precise, particularly within the training domain. However, it was also indicated that such models can have reduced responses to physical changes in the system especially when the changes occur outside the training domain or when the model is trained using steady-state data.

### Semi-empirical NO_x_ models

Simplified chemical kinetics based semi-empirical models are computationally inexpensive and with some initial tuning, can yield accurate NO_x_ predictions across a wide range of operating conditions.^10^ These models commonly assume that NO production takes place predominantly in the mixing-controlled (diffusion) combustion phase and that the major production pathway is the thermal (Zeldovich) mechanism.^25,26^ Crank-angle resolved cylinder-pressure is used to determine the thermal state of the combustion mixture and the characteristic thermal NO production temperature.^7,10,12^ Adiabatic flame temperature $\left(T_{\mathrm{ad}}\right)$ is commonly used as the characteristic NO production temperature^7,10,27^ as it is considered to be a good estimate of the NO production temperature of a quasi-steady diffusion flame.^12,26^ Because cylinder pressure sensors are expensive and not found in production engines, some semi-empirical models estimate the cylinder pressure indirectly from fuel injection data.^28,29^

NO_2_ can form a significant (up to 30%^30^) fraction of the total engine-out NO_x_ emissions from diesel engines, particularly at higher levels of EGR, but to avoid increasing model complexity, semi-empirical models usually do not explicitly estimate NO_2_. They assume that NO_2_ emissions are either negligible or proportional to NO emissions, which can be reconciled during model calibration using experimental NO_x_ data. Some semi-empirical models, however, have additional mechanisms to estimate NO_2_.^29,30^

Semi-empirical models are typically calibrated using steady-state engine data (static tuning) and then their performance is tested at other steady-state points^7,11,12^; or for models being developed for real driving / transient control, additional testing is conducted over different driving cycles. If needed, this is followed by dynamic model tuning using NO_x_ emissions data collected from on-board or portable emissions analysers.^28,29,31,32^ Such real driving NO_x_ emission sensors, however, can be limited by their measurement accuracy (10%–12% errors^6,29^).

Lyons and Timoney^10^ used the simplified Zeldovich mechanism with $T_{\mathrm{ad}}$ to estimate NO_x_ emissions. Then using a quadratic formulation, they estimated actual NO_x_ emissions by regressively fitting the predictions to measured values from 521 steady-state test points. They reported good model performance (*R*^2^ over 98%). Arrégle et al.^27^ assumed that NO production was proportional to the instantaneous apparent heat release rate (AHRR). The model did not explicitly estimate Zeldovich NO but, instead, used $T_{\mathrm{ad}}$ , AHRR and engine speed in an exponential NO_x_ production equation. Engine speed served as a proxy for in-cylinder mixing and turbulence levels. Three tuning parameters were calibrated using a fitting algorithm. Guardiola et al.^12^ modified the Arrégle et al. model by additionally accounting for ‘re-burning’ (the re-entrainment of NO in the reducing zone of a diffusion flame), which improved the prediction accuracy by 1.3% at a slight computational time penalty.

Park et al.^7^ used CFD simulated NO production results at 35 different steady-state operating conditions to develop a physical model. The model used $T_{\mathrm{ad}}$ and an effective NO production window to compute cyclic NO emissions. It had only one tuning parameter (Zeldovich pre-exponential coefficient) and reported overall good performance (*R*^2^ over 98%). Guardiola et al.^31^ used an exponential NO_x_ correlation with multiple correction factors (for inlet air temperature and humidity, coolant temperature and transient operation) to predict emissions during transient operation. The ‘nominal’ NO_x_ values, instead of being estimated from a Zeldovich-derived equation, were obtained from steady-state experimental calibration maps. The model produced an average error of 50 ppm during transient operation. Barbier et al.^32^ used real driving NO_x_ data from a hybrid diesel vehicle to calibrate the Guardiola et al.^31^ model, and reported mean absolute errors of around 20 ppm. They declared real driving NO_x_ emissions to be a ‘viable’ method of calibrating semi-empirical NO_x_ models. Lee et al.^29^ in their semi-empirical model, assumed that NO formation was proportional to the amount of fuel injected and reported around 10% error in transient driving NO_x_ predictions.

### Paper scope

The current study uses a modified version of the semi-empirical model by Part et al.^7^ to test the hypothesis that a physical model that uses peak cyclically-resolved cylinder pressure to predict cyclic NO_x_ emissions will have good transient performance. This hypothesis is based on high-speed (cyclic) NO_x_ emissions measurement studies that have reported strong linear correlations between peak cylinder pressure and NO_x_ emissions.^21,33–35^ The modified physical model instead of using CFD-predicted cylinder pressure uses experimental cylinder pressure data for 1800 consecutive cycles with cyclic, transient exhaust NO_x_ data (measured using advanced ‘fast’ NO and NO_2_ analysers) for model development and testing. Moreover, it directly computes thermochemical properties of the combustion mixture using the open source chemical-kinetics and equilibrium solver *Cantera*,^36^ instead of using correlations developed for a limited range of mixture conditions.

The paper also presents an alternate (to using portable or on-board NO_x_ sensors) means of testing and dynamically tuning semi-empirical transient NO_x_ prediction models by using high accuracy fast exhaust NO_x_ measurements.

## Methodology

### Experimental setup

**Figure 1.**
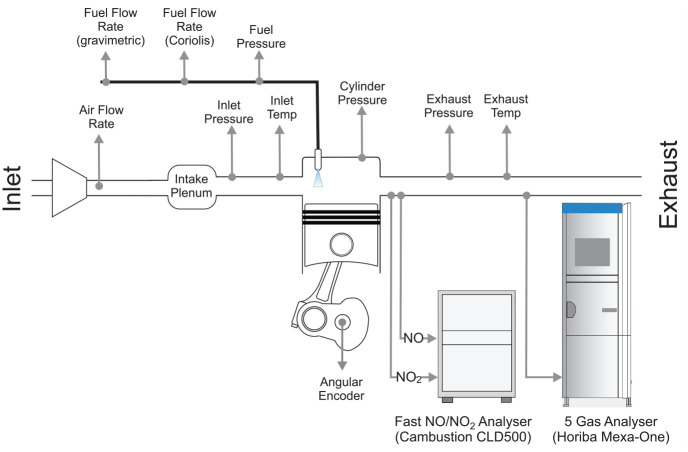
Schematic of experimental setup with relevant sensors labelled.

Experimental data was collected from a single-cylinder, automotive-type, compression-ignition (diesel) research engine. The engine and the test setup have been described in detail in previous publications.^21,37^ Important specifications of the engine are summarised in Table 1 and a schematic of the setup with relevant sensors is shown in Figure 1.

**Table 1.** Engine specifications.

Bore [mm]	83.0
Stroke [mm]	92.4
Displacement [cm^3^]	500.0
Valves per cylinder	2 intake, 2 exhaust
Fuel injector	8-hole solenoid direct injector

Cylinder pressure was measured every 0.1 CA° using a *Kistler 6046Asp-3-2* transducer, and air and fuel flow rates were recorded at 1 Hz. A *Sierra CP Airtrak 620S* flow sensor was used for air flow measurement and fuel flow was measured independently using a gravimetric (*Sierra CP FuelTrak*) and Coriolis (*Siemens MASS2100*) flow meter. The engine was run on a standard EN590 compliant diesel fuel.

Exhaust NO_x_ emissions were measured using a standard (‘slow’) *Horiba MEXA-ONE* analyser, and ‘fast’ NO and NO_2_ analysers manufactured by *Cambustion*. The *Horiba MEXA-ONE* worked on the chemiluminescence principle to measure NO and NO_x_ concentrations. Its NO channel had a response time of 1.2 s while the NO_x_ channel had a response time of 1.5 s. However, because of transport delays due to the exhaust sampling point being significantly downstream of the exhaust ports (after an exhaust backpressure valve and a smoothing tank), the effective response time for the analyser was 15 s.^37^ The fast NO analyser (*Cambustion CLD500*) also worked on the chemiluminescence principle but used a constant pressure heated sampling chamber to have a low $T_{10-90\%}$ response time 0.2 ms. The fast NO_2_ analyser used laser-induced fluorescence with a heated capillary gas sampling system to achieve a high response rate of $T_{10-90\%}$ = 0.2 ms.^38^ Exhaust samples for the fast NO_x_ analysers were collected approximately 70 mm downstream of the exhaust port. Special care was taken to ensure that measurement errors were kept low, including recalibrating the fast NO_x_ analysers every hour to account for any calibration drift. The raw signals from the fast NO analyser were also corrected for any quenching that could have occurred in the chemiluminescence detector.^37^

**Figure 2.**
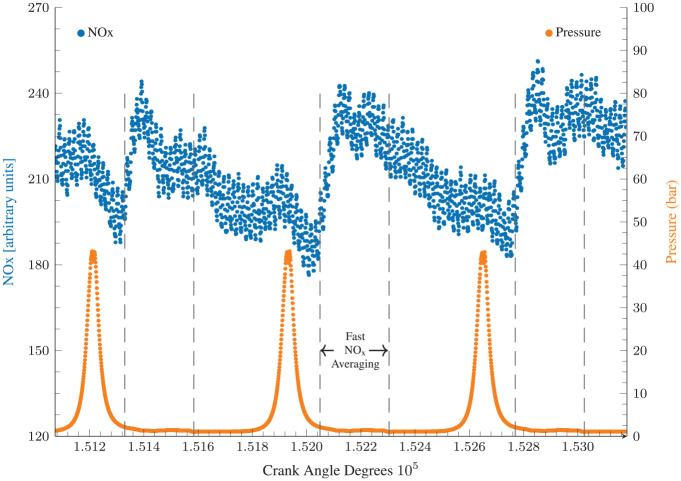
Crank angle resolved fast NO_x_ data and the corresponding crank angle resolved pressure measurements. Sample crank angle resolved NO_x_ averaging windows from EVO to EVC (dashed) for cyclic NO_x_ calculations.

As discussed in Fang et al.,^15^ slow and high speed data, which were recorded every 1 s and 0.1 CA°, respectively, were time-aligned to account for transportation delays. Crank-angle resolved NO_x_ data was used to calculate cyclic NO_x_ emissions by averaging NO_x_ concentration during the exhaust valve open period. Figure 2 shows sample NO_x_ results from the fast analyser for three consecutive cycles, along with corresponding cylinder pressure data to illustrate the NO_x_ averaging window from exhaust valve opening (EVO) to exhaust valve closing (EVC). This period was selected as the fast NO_x_ signals are valid only during this period when combustion products from the cylinder are being expelled. A mean value is then calculated over the valve opening period which represents NOx emissions for that cycle. Ball et al.^39^ used a similar averaging approach and reported close (greater than 95% for most cases) agreement with mass averaged high speed NO measurements.

Owing to data confidentiality requirements, all emissions data reported here has been re-scaled by an arbitrary scaling factor and thus presented in ‘arbitrary units’.

### Operating conditions

Data from two transient engine operating conditions, summarised in Table 2, was used for model calibration and testing. Transient engine operation was achieved on the engine dynamometer by adjusting fuel injection duration to switch between low and high load operating points, whilst maintaining other combustion parameters such as location of 50% mass fraction burned constant. High and low speed data were continuously collected for at least 1800 cycles at each condition. The first operating condition (Condition 1) represents low-load, medium-speed transient operation while Condition 2 represents high-load, high-speed transient operation. For both operating conditions, alternation load steps are applied to represent real driving conditions.

**Table 2.** Experimental test conditions used for model development.

	Condition 1	Condition 2
Engine speed [RPM]	1500	2000
Lower nIMEP [bar]	1.9	19.4
Higher nIMEP [bar]	3.8	25.8
Back-pressure [barG]	0.31	2.9
Inlet temperature [°C]	55	40
EGR [%]	0	0

### Physical model

A physical model was adapted from the model of Park et al. ^7^ The model assumed that NO was produced only during diffusion combustion and was formed via the thermal (Zeldovich) pathway, which is active at temperatures above 1800 K.^40^ The simplified thermal NO production rate equation, equation (1),^25^ which is based on steady-state and equilibrium assumptions applied to the extended Zeldovich mechanism (Reactions R1 to R3) was used. In equation (1), *T* is the absolute temperature, terms within square brackets are molar concentrations, subscript *e* denotes equilibrium concentrations, *A* is an empirical pre-exponential factor, and *B* is an activation energy index.

(1) $$\frac{d[NO]}{dt}=\frac{A}{\sqrt{T}}\exp(\frac{B}{T}){{\left[O_{2}\right]}_{e}}^{\frac{1}{2}}{\left[N_{2}\right]}_{e}$$

(R1) $$O+N_{2}=\mathrm{NO}+N$$

(R2) $$N+O_{2}=\mathrm{NO}+O$$

(R3) N+OH=NO+H

Peak thermal NO production rates were used as a proxy for average NO production rates as both were found to be proportional by Park et al.^7^ Crank-angle resolved measured cylinder pressure, along with calculated trapped cylinder mass and volume, were used to determine average cylinder temperature. Trapped cylinder mass was calculated from measured air and fuel flow rates by assuming a constant residual gas fraction, which represents the fraction of gases retained from the preceding cycle, that is, ‘internal EGR’.

For our study, $T_{\mathrm{ad}}$ (adiabatic flame temperature) was assumed to be the characteristic NO production temperature and was calculated at the start of combustion (SoC), taken to be the 10% mass fraction burned (mfb) point, using *Cantera*. N-heptane was selected as the single component chemical surrogate to represent the diesel fuel. A constant-volume batch reactor was used to simulate $T_{\mathrm{ad}}$ in *Cantera* where a 68-species skeletal mechanism from Lu et al. for n-heptane is used.^41^ Then the temperature at the peak pressure point $\left(T_{\max}\right)$ was calculated using equation (2) by assuming that the burned gas was compressed isentropically during combustion. $T_{\max}$ was computed for each cycle and was used to calculate the peak cyclic NO production rate from equation (1). Equilibrium O_2_ and N_2_ concentrations were also obtained from *Cantera*.

(2) $$T_{\max}=T_{\mathrm{ad}(\mathrm{SoC})}\left(\frac{P_{\max}}{P_{\mathrm{SoC}}}\right)$$

**Figure 3.**
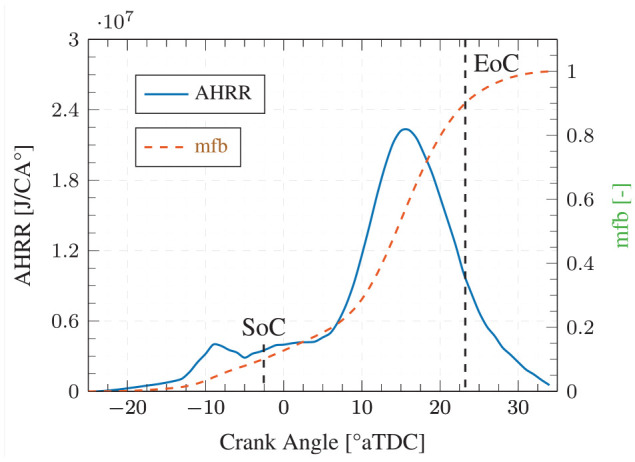
Combustion and effective NO_x_ production windows for a selected cycle.

Cyclic NO emissions were then calculated using the peak NO production rate and an effective NO production period of 10%–90% mfb illustrated in Figure 3. Since the highest NO production rates in diesel flames have been observed in the stoichiometric regions,^26^ an equivalence ratio (ϕ) of 1 was used for thermal NO production calculations.

NO_x_ estimates were obtained from the NO predictions by assuming a constant NO_2_:NO_x_ ratio. From the high-speed NO and NO_2_ measurements averaged for all the recorded cycles, conditions 1 and 2 had NO_2_:NO_x_ ratios of 0% and 10%, respectively, which were used in the physical model. The high NO_2_:NO_x_ at condition 2 are believed to result from the sporadic phenomenon of ‘injector dribble’ which was more active at the high load condition. Hydrocarbons introduced to the reacting mixture by injector dribble can generate HO_2_ radicals and oxidise some of the NO to NO_2_ as per Reaction R4. This was investigated in previous studies on the same engine using high-speed exhaust NO, NO_2_ and hydrocarbon sensors.^35,38^ A strong, linear correlation $\left(R^{2}>0.8\right)$ between cyclic exhaust hydrocarbon and NO_2_ was observed under similar conditions.

(R4) $$\mathrm{NO}+{\mathrm{HO}}_{2}={\mathrm{NO}}_{2}+\mathrm{OH}$$

A simple net apparent heat release formulation shown in equation (3) that assumed a constant ratio of specific heats (γ) was used to determine the mfb profile.^25^ Guardiola et al.^12^ showed that using a more comprehensive AHRR model increased computational time by more than 200 fold. Therefore the choice of the simple AHRR model was considered appropriate.

(3) $$\frac{dQ_{\mathrm{net}}}{d\theta }=\frac{\gamma }{\gamma -1}P\frac{dV}{d\theta }+\frac{1}{\gamma -1}V\frac{dP}{d\theta }$$

**Figure 4.**
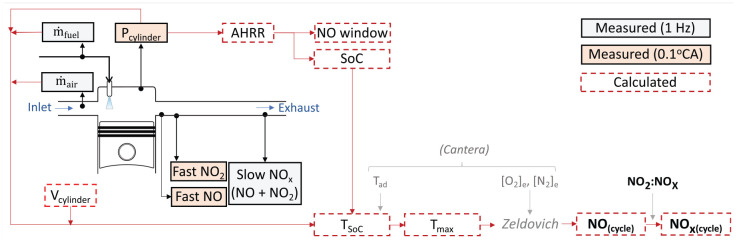
Overview of the physical NO_x_ model’s architecture.

The model’s architecture is summarised in Figure 4 and a summary of the important parameters used in it and their sources is provided in Table 3.

**Table 3.** Physical NO_x_ model parameters.

Parameter	Source/Value
*Cylinder pressure*	Measured every 0.1 CA°
*Air and fuel flow rate*	Measured at 1 Hz
*Fast $NO_{x}$ *	Measured every 0.1 CA
*Cyclic $NO_{x}$ *	Fast $NO_{x}$ averaged from EVO to EVC
*Slow $NO_{x}$ *	Measured at 1 Hz
*Cylinder volume*	Calculated from kinematics
$[O_{2}{]}_{e}$ , $[N_{2}{]}_{e}$	Calculated from *Cantera*
*Residual gas fraction*	12% (condition 1), 6% (condition 2)
ϕ	Assumed stoichiometric
γ	1.3
*Start of combustion*	10% mfb
*NO production window*	10%–90% mfb
$T_{\mathrm{ad}}$	Calculated from *Cantera*
*Zeldovich parameter B*	Constant value of -69090^7^
*Zeldovich parameter A*	Tuned (final value: $9.8\cdot 10^{16}$ )
*NO*_2_:*NO*_x_	0% (condition 1), 10% (condition 2)

### Neural network model

**Figure 5.**
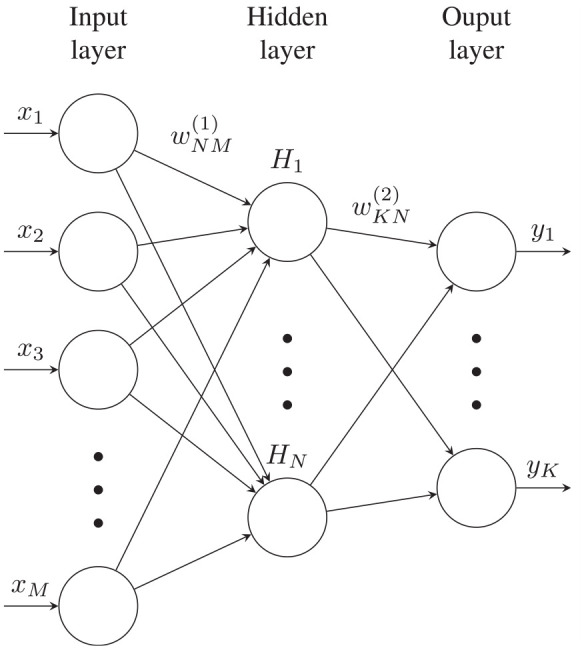
Schematic diagram of the neural network structure used as comparison.

The artificial neural network model used for comparison has been described in detail in previous publications.^15,21^ It was based on a common ANN structure known as a multilayer perceptron illustrated in Figure 5, and comprised one input layer, one hidden and one output layer. The input parameters were carefully chosen through different filtering approaches where 14 engine parameters were identified and used as the input layer. A log-sigmoid activation function with a mean squared error (MSE) based error function was used. While the use of multiple-layer structure is adapted in a lot of studies centring around NO_x_ prediction, for the conditions tested in this study one-layer structure showed very high accuracy for all conditions tested. It is to be noted that the conditions studied here were never used or included in both the training and validation processes during the construction of the neural network. In addition, no fast NO_x_ results and maximum cyclic pressure were ever used during the training of the neural network. The neural network training was purely based on steady-state slow NO_x_ data. The predictions from ANN demonstrated in this study as a comparison therefore are mostly based on extrapolation where cyclic maximum pressure and other 13 channels of repopulated data are together used as the input. It is to be noted that the emphasis of this study is not on the machine learning aspect, but rather on the development of the semi-empirical NOx model, which holds greater relevance in this context. Nevertheless, we believe it is important to include previous results of a simplified machine learning model, considering it was trained using the same dataset. While the prior paper on machine learning primarily dealt with the impact of engine parameter selection, rather than the development of the ANN model itself, its inclusion in this study is justified. Both the semi-empirical and the machine learning models utilised maximum cylinder pressure as a key factor. This also motivates us to pursue a more physics-based approach in future machine learning model development. We also acknowledge the recent advances in ML modelling in the context of engine development which can significantly improve the current neural network model. For example, the use of hyperparameter tuning and optimization^42^ and the use of superlearner-based ensemble-ANN techniques^23,24^ can be of interest.

## Results and discussion

### Physical model calibration and testing

The model was calibrated manually for condition 1 by comparing cyclic experimental and physical model-predicted NO_x_ concentrations. This entailed selecting physically reasonable values for the Zeldovich coefficient *A* and an effective SoC timing that minimised NO_x_ prediction errors. It was found during calibration that using slightly higher $P_{\mathrm{SoC}}$ values to find $T_{\max}$ in equation (2) yielded more accurate results. Therefore, cylinder pressure slightly after 10% mfb was used as a marker for effective SoC. An effective SoC of 17.5% mfb was used in the tuned model.

**Figure 6.**
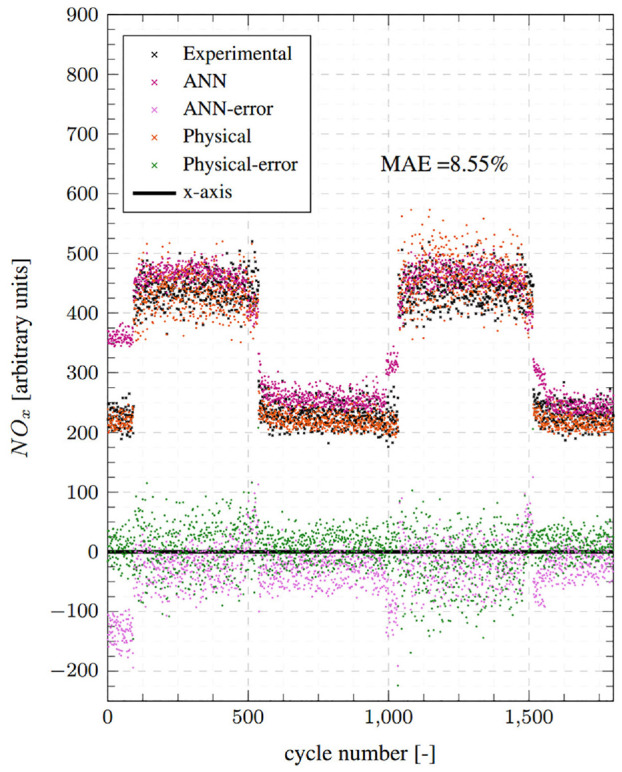
Top: Test point 1 comparisons between the physical model predictions, ANN NO_x_ model predictions and the cycle averaged fast-NO_x_ analyser readings. Bottom: Absolute errors for both ANN and physical models.

**Figure 7.**
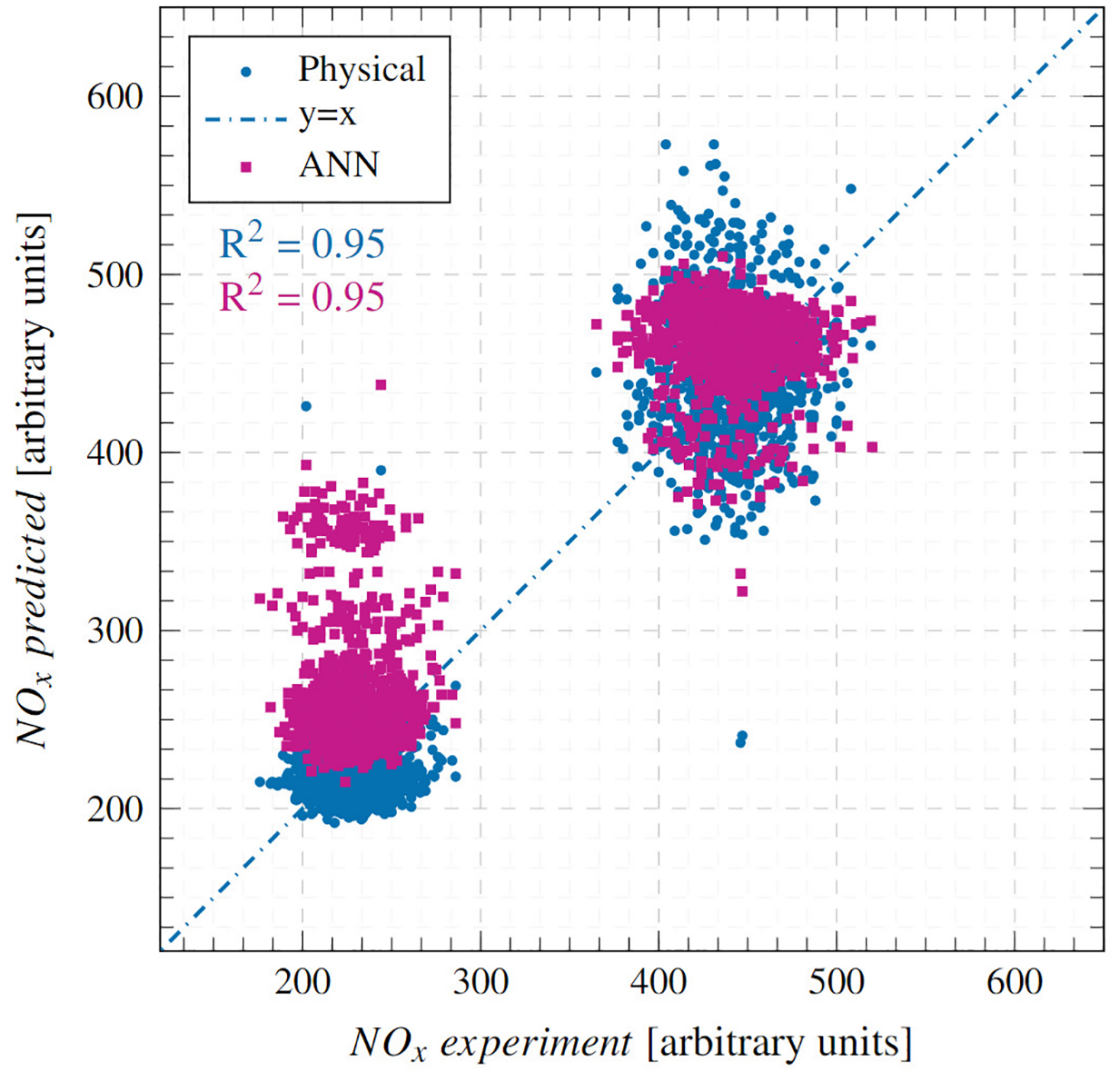
Correlation between experimental and predicted NO_x_ emissions at condition 1.

Figure 6 compares the physical model predictions with experimental values at condition 1 for the 1800 cycles considered. It also reports the cyclic errors (at the bottom) and the mean absolute error (MAE) as a percentage. Predictions from the ANN model, which will be discussed later, are also shown. The physical model demonstrates very good accuracy and transient response, whereby a low MAE of 8.55% is registered and the NO predictions track experimental values closely during the highly transient step-up/down events. Figure 7 illustrates the accuracy of the physical model on a correlation plot and reports a high goodness of fit with a coefficient of determination (*R*^2^) value of 0.95. No prominent data clusters are observed away from the y=x line. These results are encouraging as they demonstrate that a simple physical model with minimal tuning can capture cyclic level changes in NO_x_ emissions.

**Figure 8.**
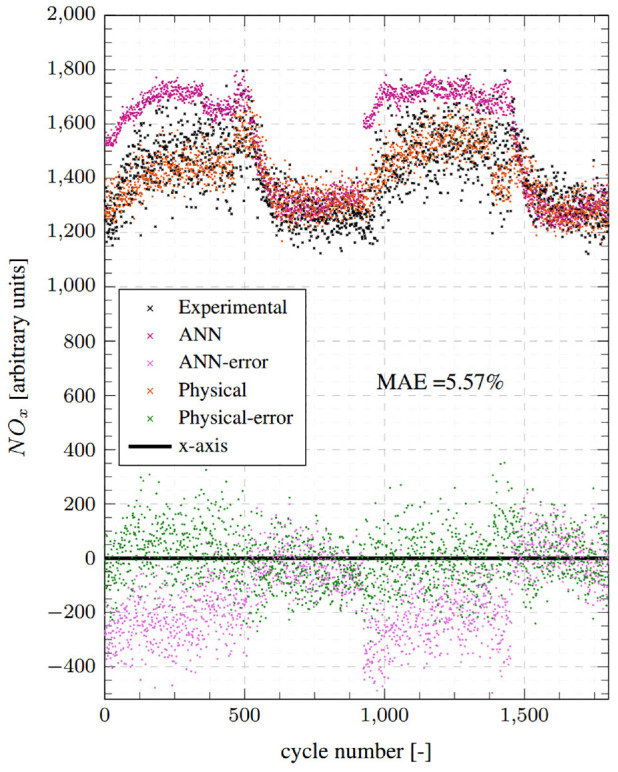
Top: Test point 2 comparisons between the physical model predictions, ANN NO_x_ model predictions, and the cycle averaged fast-NO_x_ analyser readings. Bottom: Absolute errors for both ANN and physical models.

Next, the calibrated model was tested at condition 2 without changing any parameter settings except for the residual gas fraction, which was halved from 12% to 6% – assuming that the higher load case would have lower trapped residuals.^25^ Model prediction results at this condition are presented in Figure 8. The model registers a very good transient performance by closely tracking experimental NO_x_ values and rapidly responding to load changes. The resulting errors are low with an MAE of 5.57%. The model’s performance at condition 2, which represents high load transient operation, is very encouraging for transient NO_x_ monitoring and control as the model was tuned under significantly different operating conditions at condition 1.

**Figure 9.**
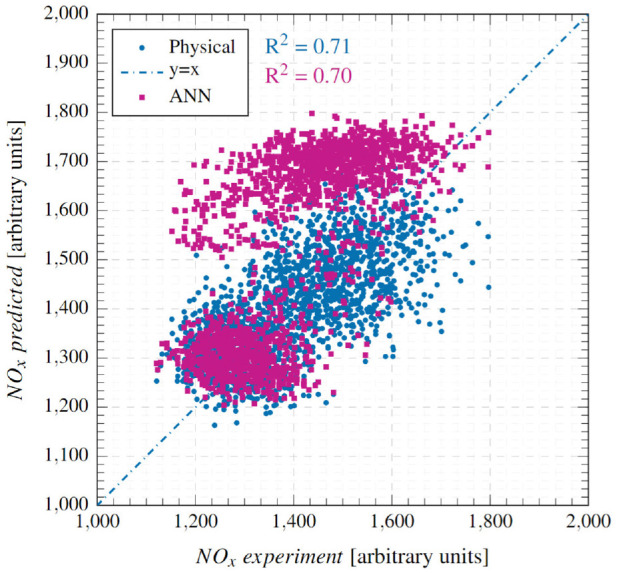
Correlation between experimental and predicted NO_x_ emissions at condition 2.

Figure 9 presents the model’s prediction results at condition 2 as a correlation plot. A strong correlation between predicted and experimental NO_x_ emissions with an *R*^2^ of 0.70 is observed. As with condition 1, no obvious clusters away from the y=x line are present, which showcases the model’s predictive strength across the entire load range swept at condition 2. However, the *R*^2^ value is lower than that at condition 1 (0.95), signifying a larger spread in the prediction results. This is an artefact of the experimental NO_x_ data at condition 2 having a higher degree of scatter. The absence of two distinct high and low load clusters in Figure 9, which were observed in Figure 7 for condition 1, illustrates this increased scatter. The high experimental scatter is primarily due to the transient load step at condition 2 being an increase of around 33% whereas at Condition 1 the increase is 100%. In addition at condition 2, some of the scatter can be attributed to the conversion of some of the NO produced during combustion to NO_2_ via injector dribble-induced reactions discussed earlier.^38^ At the relatively high load of condition 2, it is unlikely that much thermal NO_2_ will be being produced and so this hydrocarbon induced mechanism is a likely cause.

The accuracy of the physical model’s predictions is affected as a result because it only considers thermally produced NO and ignores other NO production and conversion mechanisms. The addition of sub-models for non-thermal NO_x_ production can potentially improve the results. This limitation of the model can, however, be leveraged for diagnostic purposes to identify abnormal NO_x_ production activity or sensor malfunction when scatter in NO_x_ estimates increases.

### Comparison with ANN model

The performance of the physical and the ANN models is compared by referring to the results presented above in Figures 6 to 9. Overall, the physical model is found to perform better than the ANN model, both in terms of transient and steady-state accuracy as evidenced by the consistently low error values in Figures 6 and 8. The physical model has MAEs of 8.55% and 5.57% for conditions 1 and 2, respectively, while the ANN model has errors of 12.69% and 10%. Moreover, as was highlighted in Fang et al.^15^ and can be seen from the ANN results reproduced here, the ANN model has regions of high errors during transient operation, especially during step-up events, for example, first 100 cycles of condition 1 and cycles around 1000 when the second step-up event takes place (Figure 6); and first 400 cycles and cycles 9000-1500 for condition 2 (Figure 8). These high error points in the ANN model estimates appear as clusters in the corresponding correlation plots (Figures 7 and 9) above the y=x line, signifying overestimation errors. The clustering observed in the ANN results is an artefact of its architecture and training regime, whereby steady-state data was used for its training. As mentioned earlier, the 14 input parameters used as input channels for the ANN model are repopulated with only cyclic maximum pressure used at the same frequency as the NO_x_ experiment, which can significantly contribute to poor predictions near load steps. This is further confirmed at steady regions of both operating conditions where the ANN model performed better for both conditions. It is to be noted that Condition 2 by nature can be significantly more challenging for the neural network model based on steady-state data, due to the different response times of all input channels and the fast condition changes that occurred in the testing. During high-load conditions, the discrepancies are also likely due to the fast analyser picking up the cycle-to-cycle variations which the model is not trained with. Whereas the physical models relying on cyclic peak pressure can also reflect this cycle-to-cyle variation. This was highlighted previously in our previous study.^15^ This further highlighted the importance of having high-frequency high-fidelity data as an input for neural network-based models which can be seen as a challenge in using such models for cyclic NO_x_ predictions.

The reason for the physical model’s superior response is its structure where crank angle resolved cylinder pressure data was used to capture cyclic changes in combustion intensity and phasing, and its effects on the NO production temperature. Previous high-speed NO_x_ measurement studies^33–35^ have reported strong linear correlations between peak cylinder pressure and NO_x_ emissions. Fang et al.^21^ performed the Pearson correlation test on 32 measured engine parameters and cyclic NO_x_ emissions; and identified peak cylinder pressure as the parameter with the highest importance. They also reported a high Pearson correlation coefficient score for coolant temperature change. This indirectly pointed to a strong correlation between peak cylinder temperature and NO_x_ emissions because an increase in peak cylinder temperature leads to increased wall heat transfer and coolant temperature changes.

**Figure 10.**
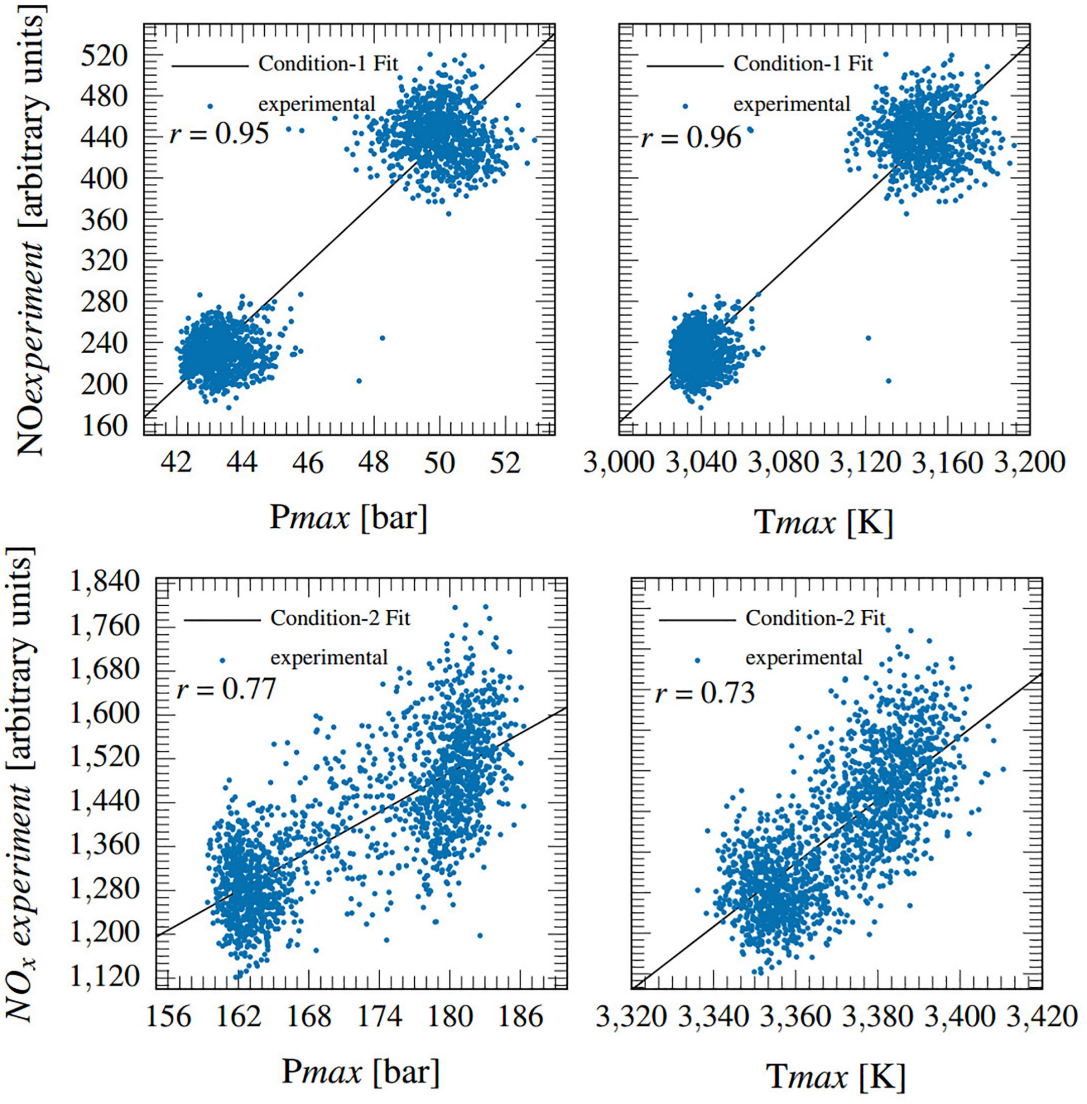
Correlation of experimental cyclic NO_x_ with (left) peak cylinder pressure and (right) corresponding cylinder temperature.

These findings are confirmed for the current data set in Figure 10 in which the left column shows the strong correlation that exists between peak pressure (measured) and exhaust NO_x;_ and the right column shows a comparably strong correlation between $T_{\max}$ (calculated from the physical model) and cyclic NO_x_ emissions. This also demonstrates the suitability of the characteristic $T_{\mathrm{ad}}$ based NO_x_ production temperature choice. It is worth noting that the predicted value correlation with the experimental NO_x_ follows the correlation highlighted in Figure 10 where Condition 1 is significantly better. This is again likely caused by injector dribble-induced reactions during the experiments.

The observed strong correlation between in-cylinder peak thermal conditions and exhaust NO_x_ concentrations can serve as a guide for developing simple (data-driven or physics-based) NO_x_ prediction models by establishing accurate estimation of peak cylinder pressure and temperature as a requirement for accurate NO_x_ predictions; and data from advanced fast NO_x_ analysers (like the ones used here) can provide cyclic level information about changes in NO_x_ emissions for dynamically tuning such models. Since the ANN model was not trained using cyclic data, it failed to accurately predict peak cylinder pressure during transient operational changes and thus had high errors during those periods as seen in Figures 6 and 8.

### Comparison with slow speed NO_x_

The slow-speed, chemiluminescence based NO_x_ analyser is commonly considered the benchmark for measurement accuracy but its slow dynamic response limits its utility for accurately measuring transient NO_x_ emissions. Therefore on-board and portable emissions analysers, which have higher measurement errors, have been used for developing transient NO_x_ models.^28,29,31,32^ It has been shown previously^37^ that measurements from fast NO_x_ analysers are in very close agreement with those from slow-speed analysers, which provides confidence in their measurement accuracy and thus makes them a source of high-fidelity transient NO_x_ data.

**Figure 11.**
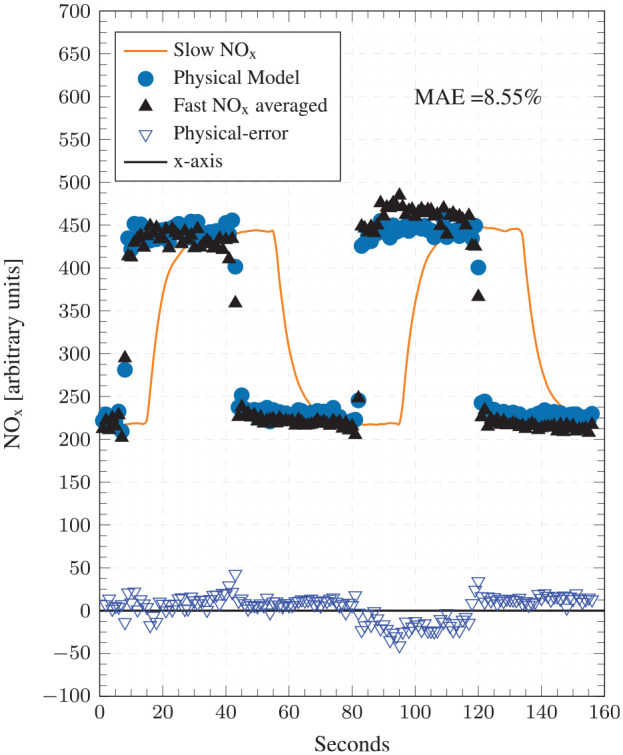
Test point 1 comparisons between the cycle averaged physical NO_x_ model predictions, the time-averaged fast-NO_x_ analyser readings and the slow-NO_x_ analyser readings.

**Figure 12.**
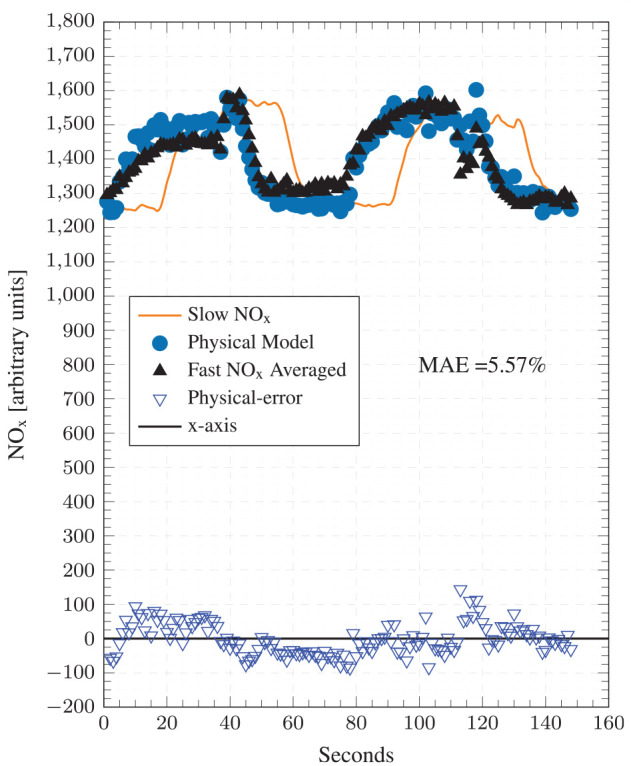
Test point 2 comparisons between the cycle averaged physical NO_x_ model predictions, the time-averaged fast-NO_x_ analyser readings and the slow-NO_x_ analyser readings.

Figures 11 and 12 compare the experimental and predicted cyclic NO_x_ (averaged over 1 s windows) to the slow speed measurements. The cyclic averaged NO_x_ measurements are carefully aligned with the slow speed measurements through other channels to ensure a fair comparison. It can be seen that step responses in the slow speed analyser readings are severely delayed, which significantly under or over-reports exhaust NO_x_ concentrations during step-up and step-down episodes, respectively. This is linked to the physical position of the slow analyser further downstream in the exhaust, and the slower rise and drop are caused by the its slower dynamic response ( $T_{10-90\%}$ of 1.5 s vs 0.2 ms). The difference in peak NO_x_ emissions between two analysers is also likely caused by the location of the analyser where the agreement between the two analysers is highlighted in previous experimental work assuring the fidelity of both measurements.^35^

The physical model was able to respond rapidly to cyclic changes in combustion and transient NO_x_ estimates from it thus closely following the fast NO_x_ measurements. For condition 1, the time-averaged physical model and the fast analyser reach steady-state almost instantly whereas the slow analyser takes around 6–8 s to reach a steady-state value after the load step, suggesting that engine-out NO_x_ emissions follow a slow transient path on a load step. Physically, a delay in the NO_x_ getting to a steady state as shown by the slow-NO_x_ channel would suggest that there is more NO measured than NO_x_, which is not an accurate representation of the NO_x_ formation process. This is also highlighted by the previous study where a close alignment of NO_x_ and NO from the fast-NO_x_ analyser suggested NO_2_/NO_x_ ratio has a similarly instantaneous response.^35^

These results demonstrate that standard slow-speed NO_x_ analysers are ill-suited for dynamic engine testing and calibration, and fast-responding, accurate models like the physical model developed here can help bridge this gap. To the best of the author’s knowledge, the fast transient NO_x_ behaviour was for the first time demonstrated and validated through a semi-empirical NO_x_ model. The sub-10% errors in the physical model estimates put such models at par with portable and on-board NO_x_ analysers.^6,29^ These models can thus serve as virtual sensors for real-time emissions monitoring to ensure RDE compliance. And perhaps can also be served as part of the model structure in physics-guided neural networks for NO_x_ emission predictions.^43^

## Conclusions

A simple physical model was developed in this study where the calibration was completed against crank-angle resolved NO_x_ emissions data measured from a high-speed diesel engine undergoing transient operation. The model was tested at a different transient operating point and produced acceptably accurate (MAE = 5.57%) NO_x_ emissions estimates. The model’s transient performance was found to be better than that of a previously developed ANN-based model. This improvement was attributed to the physical model’s design, whereby characteristic NO_x_ production temperature calculated for each cycle from crank-angle resolved cylinder pressure data served as a dynamic NO_x_ predictor. However, at operating points where non-thermal NO production / conversion mechanisms were present (injector dribble induced NO_2_ production in the current study), the physical model’s accuracy suffered.

It was also shown that standard slow-speed NO_x_ analysers were ill-suited for dynamic engine testing and calibration, and fast-responding, accurate models like the physical model developed here can help bridge this gap. It was also for the first time demonstrated that a simple semi-empirical NO_x_ model based on cyclic peak pressure can indeed better capture the fast transient NO_x_ behaviour compared to slow-speed analysers. In the future, results from the physical model can be used to guide the training of a machine learning model, that is, a hybrid model, to improve its predictions at transient conditions. Moreover, the various NO_x_ prediction models can be tested at more extensive transient operating conditions to compare their performance under real-world driving scenarios.

## Abbreviations

${\left[N_{2}\right]}_{e}$ Equilibrium nitrogen concentration

${\left[O_{2}\right]}_{e}$ Equilibrium oxygen concentration

γ Ratio of specific heatsz

ϕ Equivalence ratio

θ CA location

A, B Zeldovich coefficients

AHRR Apparent heat release rate

ANN Artificial neural network

aTDC After top dead centre

CA Crank angle

CoV Coefficient of variation

EGR Exhaust gas recirculation

EVC Exhaust valve closing

EVO Exhaust valve opening

IMEP Indicated mean effective pressure

MAE Mean absolute error

mfb Mass fraction burned

MSE Mean square error

P Pressure

RDE Real driving emissions

RPM Revolutions per minute

SoC Start of combustion

T Temperature

t Time

T_ad_ Adiabatic flame temperature

V Volume
